# Non-coding transcriptome in brain aging

**DOI:** 10.18632/aging.101290

**Published:** 2017-09-12

**Authors:** Thomas Arendt, Uwe Ueberham, Michael Janitz

**Affiliations:** Paul-Flechsig-Institute for Brain Research, University of Leipzig, Leipzig, Germany

**Keywords:** long non-coding RNAs, circular RNAs, brain, transcriptome, RNA sequencing, neurodegenerative diseases

Decreasing cognitive function with different degrees of severity affects most of the aged population. A decline in cognitive abilities may be the only symptom of aging in otherwise healthy people. Along with age-related neurodegeneration, the cognitive decline represents a significant burden for society and the health system. The etiology of age-related cognitive decline remains still poorly understood. As a result, the development of targeted therapeutics that are designed to prevent or reverse the loss of cognitive function in aging individuals has proved to be difficult, and there are currently few effective treatments for age-related cognitive impairments [1].

A rapidly growing number of studies clearly show that the intergenic regions of the human genome exhibit tissue-specific transcription. This so-called pervasive transcription results in the production of a variety of non-coding RNA species, including long non-coding RNA (lncRNA) transcripts (i.e. greater than 200 bp), which are involved in a wide range of structural, regulatory, and catalytic processes. LncRNAs are transcribed in complex intergenic, overlapping, and antisense patterns relative to adjacent protein-coding genes, suggesting that many of the RNAs regulate the expression of these genes.

Important phenotypic changes that have occurred during human evolution, particularly between humans and other primates, stem from elevated levels of gene transcription observed in these species. Indeed, major evolutionary changes may have occurred on the transcriptomic level and they appear to be particularly evident for long non-coding RNAs (lncRNAs). Genomic surveys of a wide selection of species have clearly shown the relative number of the non-coding sequences increases consistently with organism complexity. Thus, lncRNAs are most likely to be a critical component of gene regulation in complex organisms that gained a san increasing significance during evolution of species [2].

We recently determined local transcriptome changes in synaptosome fractions from young and aging mice using RNA sequencing technique [3]. This study provides, for the first time, insights into age-related changes in gene expression profiles that encode proteins and non-coding transcripts with unprecedented resolution. Our comparative analysis revealed that the majority of the differentially expressed genes, involved in the aging process within the synapses, are novel and their RNA products do not have protein coding capacity. In addition, a significant proportion of these unannotated genes was identified as putative lncRNAs, an RNA species previously identified as important regulatory molecules in the maintenance of neuronal functionality [3].

Circular RNAs (circRNAs) are the products of backsplicing, in contrast to the canonical splicing of linear RNAs. CircRNAs are covalently closed loop structures and thus lack 5' caps and 3' poly (A) tails. Current transcriptome sequencing studies clearly show that circRNAs are expressed by 20–30% of human genes and are particularly enriched in the brain [4]. In addition, there are indications that circRNAs accumulate with the aging of the central nervous system [5]. We have recently shown that circRNAs are differently expressed in the multiple system atrophy (MSA) brain and potentially may serve as biomarkers for this disease. Our analysis of the transcriptome data from the frontal cortex of MSA and control brains, followed by RT-qPCR validation, identified five circRNAs that were statistically significant in MSA tissues. Furthermore, overexpression of the five circular transcripts was revealed in the white matter of the MSA frontal cortex compared to gray matter samples from the same brain area [6]. Indeed, circRNAs may have control over aging dependent pathways. Given that the identity of circRNAs has just recently began to be explored, we currently have only a small grasp of the role that circRNAs play in disease onset and aging. Further efforts are clearly warranted to fully elucidate the biology of these unique non-coding RNAs in the body.

In conclusion, non-coding genes, still largely unexplored, play an important role in brain biology and molecular pathology of neurodegeneration. Predominant expression and abundance of lncRNA in the human brain [7] suggests that the recent adaptive evolution of *Homo sapiens* may be critically involved in molecular pathology of neurodegeneration. Arguably, uniqueness of the human transcriptome complexity may imply an inherent limitation of the existing animal models in studying neurological disorders specific for human brain such as Alzheimer's disease.
